# Polyunsaturated Aldehydes from Large Phytoplankton of the Atlantic Ocean Surface (42°N to 33°S)

**DOI:** 10.3390/md12020682

**Published:** 2014-01-27

**Authors:** Ana Bartual, Néstor Arandia-Gorostidi, Andrés Cózar, Soledad Morillo-García, María Jesús Ortega, Montserrat Vidal, Ana María Cabello, Juan Ignacio González-Gordillo, Fidel Echevarría

**Affiliations:** 1 Department of Biology, University of Cádiz, Avda. República Saharaui s/n, Puerto Real 11510, Cádiz, Spain; E-Mails: andres.cozar@uca.es (A.C.); soledad.morillo@uca.es (S.M.-G.); nacho.gonzalez@uca.es (J.I.G.-G.); fidel.echevarria@uca.es (F.E.); 2 Instituto Español de Oceanografía, Centro Oceanográfico de Gijón, Avda. Príncipe de Asturias, 70 bis, Gijón 33212, Spain; E-Mail: nestor.arandia@gi.ieo.es; 3 Department of Organic Chemistry, University of Cádiz, Avda. República Saharaui s/n, Puerto Real 11510, Cádiz, Spain; E-Mail: mariajesus.ortega@uca.es; 4 Department of Ecology.University of Barcelona, Av. Diagonal 643, Barcelona 08028, Spain; E-mail: montsevidal@ub.edu; 5 Department of Marine Biology and Oceanography, Institut de Ciències del Mar (CSIC), Passeig Marítim de la Barceloneta, 37-49, Barcelona E-08003, Spain; E-Mail: amcabello@icm.csic.es

**Keywords:** oxylipins, diatoms, biogeographical provinces, Atlantic Ocean, polyunsaturated aldehydes, heptadienal, decadienal, octadienal

## Abstract

Polyunsaturated aldehydes (PUAs) are organic compounds mainly produced by diatoms, after cell wounding. These compounds are increasingly reported as teratogenic for species of grazers and deleterious for phytoplanktonic species, but there is still scarce information regarding concentration ranges and the composition of PUAs in the open ocean. In this study, we analyzed the spatial distribution and the type of aldehydes produced by the large-sized (>10 μm) phytoplankton in the Atlantic Ocean surface. Analyses were conducted on PUAs released after mechanical disruption of the phytoplankton cells, referred to here as potential PUAs (*p*PUAs). Results show the ubiquitous presence of *p*PUA in the open ocean, including upwelling areas, as well as oligotrophic gyres. Total *p*PUA concentrations ranged from zero to 4.18 pmol from cells in 1 L. Identified PUAs were heptadienal, octadienal and decadienal, with heptadienal being the most common (79% of total stations). PUA amount and composition across the Atlantic Ocean was mainly related to the nitrogen:phosphorus ratio, suggesting nutrient-driven mechanisms of PUA production. Extending the range of trophic conditions considered by adding data reported for productive coastal waters, we found a pattern of PUA variation in relation to trophic status.

## 1. Introduction

Phytoplankton species produce a plethora of bioactive compounds [1]. A group of these chemicals are the polyunsaturated aldehydes (PUAs), an oxylipin-type compound commonly produced by marine diatoms [2]. PUAs from diatoms have been found to be highly reactive [3], but the nature and ecological significance of these compounds remain largely unknown.

PUAs are produced after lipoxidation of intracellular polyunsaturated fatty acids (PUFAs), mainly C16 and C20, once the cell membrane has been damaged [4,5,6]. In nature, processes producing the disruption of phytoplankton cells are viral infection, grazing or/and cell lysis during senescence [7,8]. As cells die, its content and derived compounds, such as these PUAs, are released into the surrounding medium. In the vicinity of the broken cell, the released PUAs create microzones, where they react with other dissolved chemicals and interact with the neighboring organisms.

To date, different types and grades of interactions of PUAs have been described, and different ecological roles of these compounds have been proposed. Released PUAs have been suggested as a chemical defense in diatoms against grazers [2,3]. Likewise, deleterious effects on different plankton species have also been reported [9,10,11,12]. On the other hand, several studies dispute the effects of PUAs on grazers [13,14]. Recently, experiments in mesocosm studies challenged the PUA effects on bacterial community structure or diversity [15], although a strain-dependent effect has been also reported for isolated oceanic bacteria [16]. Vardi *et al.* [17] provided evidence for a PUA-derived nitric oxide-based intercellular signaling system for the perception of stressed cells, potentially playing a role in the community succession of diatom blooms. These authors proposed that as conditions become increasingly detrimental during the final phase of the bloom, the PUA concentration exceeds a threshold value and could act as a bloom termination signal, triggering cell death.

Despite the evidence implicating the effects of PUAs at different ecological levels, basic questions regarding the natural distribution of PUA producers and the range of PUA concentrations in nature still remain open. Studies in nature have been restricted to coastal areas [18]. It has been reported that octadienal, heptadienal and octadienal seems to be the most common of the PUAs produced by diatoms in culture [19] and the most commonly observed in natural blooming events [18]. Dissolved PUAs quantified after a bloom event of *Skeletonema marinoi* in the northwestern Adriatic Sea [18] ranged from 0.05 nM to 128 nM, and potential PUA levels (*p*PUA), that is, PUA concentrations obtained by artificial cell disruption of phytoplankton, reached 18.1 nmol per cell (>1.2 μm) from 1 L [18]. These observed ranges of *p*PUA in nature are very far from the micromolar concentrations usually employed in experimental toxicity assays [10], where biomass concentrations exceed natural phytoplankton abundance by several orders of magnitude. As has been pointed out by others authors [20], further analysis of natural plankton samples under non-blooming conditions will help us to understand the fluctuations and rate of production of these chemicals in the ocean.

In this work, we analyze the *p*PUA derived from large phytoplankton in the nutrient-poor open ocean. The potential PUA concentrations were obtained by mechanical cell disruption of large-sized class phytoplankton (>10 μm) collected from the surface waters (0–3 m) of the Atlantic Ocean. In an ecological context, the levels of *p*PUA must be considered as the dissolved PUA concentration that would be released in 1 L of seawater at the sampled location if 100% of the collected phytoplanktonic cells were wounded naturally (e.g., by grazers, pathogens or lysis). A large phytoplankton fraction (>10 μm) is generally composed of eukaryotic phytoplankton, mostly diatoms, dinoflagellates and coccolithophorids. They usually represent a minority fraction of the phytoplankton biomass in the open ocean, but the large phytoplankton and, especially, diatoms appear to play a more important role as oceanic productivity increases [21,22]. Given the existing knowledge about PUA production in eutrophic coastal waters [18], here, we aim to examine the nature and amount of *p*PUA derived from large phytoplankton over a range of oceanic conditions.

## 2. Results and Discussion

### 2.1. Study Site

**Figure 1:**
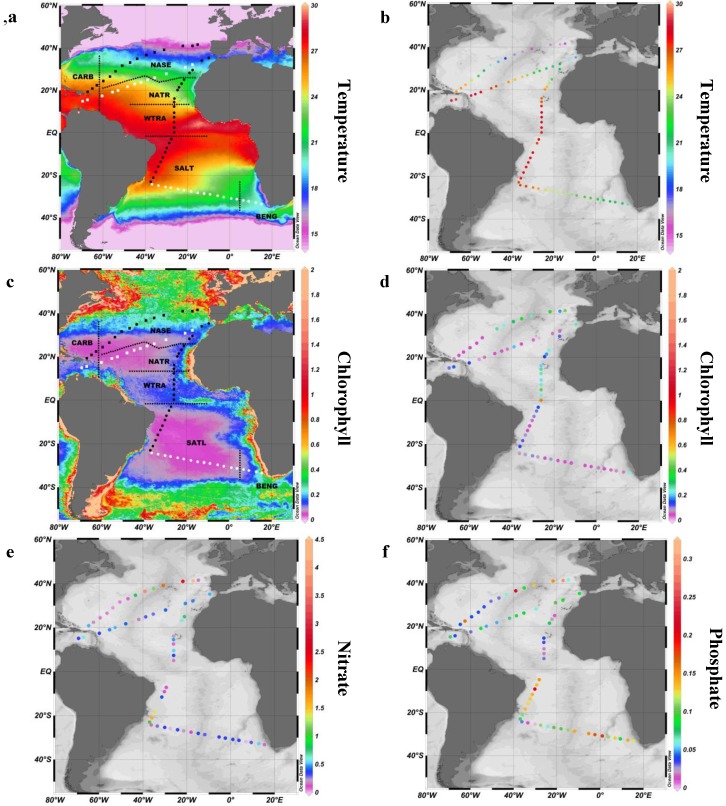
(**a**) Average temperature (°C) map in the global oceans from the sampling period (December 2010 to June 2011); (**b**) *In situ* measurements of sea surface temperature (SST) (°C). (**c**) Average chlorophyll concentration (mg m^−3^) map in the global oceans from the sampling period (December 2010 to June 2011). The NASA images are based on MODIS data from the GSFC Ocean Color team [24]; (**d**) *In situ* measurements of total chlorophyll (mg m^−3^). (**e**) *In situ* measurements of nitrate (μM). (**f**) *In situ* measurements of phosphate (μM). The location of the sampling sites are indicated over the satellite image maps with the assignment of Longhurst biogeographical provinces. Different symbols denote different transects (T1, ■; T2, □; T3, ●; T4, ○). CARB, Caribbean Province; NASE, North Atlantic Subtropical Gyre east; NATR, North Atlantic Tropical Gyral Province; WTRA, Western Tropical Atlantic; SATL, South Atlantic Gyral Province; BENG, Benguela upwelling. See Table 1 for details. The latitudinal variability of SST during the cruises is graphed in Supplementary Figure S1.

The cruises transected widely different areas in the Atlantic Ocean, embracing a range of environmental gradients in the open ocean ecosystem. Physicochemical characteristics of water masses (temperature, salinity, nutrients) ultimately determine the composition, size structure and cell physiological state of the phytoplankton community. Longhurst [23] proposed a subdivision of the ocean based on the above-mentioned water characteristics. This biogeographical regionalization of the Atlantic Ocean constitutes a useful tool for describing and comparing the *p*PUA distributions obtained in this wide sampling area. Following Longhurst’s criteria, the various biogeographical provinces were identified for the four cruise tracks (Figure 1, Table 1). Thus, latitudinal northern transects (T1 and T2 in Figure 1) visited mainly two biogeographical provinces: Caribbean Province (CARB) and North Atlantic Subtropical Gyre (NASE), passing through Sargasso Sea and the Azores region. The Sargasso Sea, with extremely low nutrient availability, denoted the boundary between the eastern and western section of this transect by defining a strong trophic gradient (Table 1). The meridional transect (T3 in Figure 1) began in Cádiz (Spain) and went southward to Brazil, travelling through NASE, the North Atlantic and the Western Tropical provinces (NATR; WTRA) and the South Atlantic Gyral Province (SATL). This transect toured most of the ultraoligotrophic ocean and crossed the equatorial upwelling. Finally, the southern latitudinal transect (T4 in Figure 1) also visited the tropical South Atlantic oligotrophic gyre and delved into the Benguela upwelling region **(**BENG**)**. In the South Atlantic Gyre, chlorophyll values are generally low throughout the year, with patches of higher phytoplankton concentrations inflowed from Benguela between October and December [23].

Our cruises sampled along a latitudinal gradient of surface temperature, with maximum temperature (>27 °C) at equatorial stations (Figure 1). The thermal gradient was especially evident in transects T1 and T2 (Figure 1), ranging from 14 °C to 27 °C. Stations sampled in the summer (T2, T4 and the southern part of T3; Supplementary Table S1) showed relatively warm waters (Supplementary Figure S1 and Table S1).

Field measurements of total chlorophyll *a* (TChl*a*) matched the pattern derived from MODIS (Figure 1; Table 1). As expected, low TChl*a* (averaged: 0.137 ± 0.125 mg m^−3^) was found in our samples. The lowest TChl*a* concentrations were found in the North Atlantic (0.066 ± 0.028 mg m^−3^) and South Atlantic (0.070 ± 0.064 mg m^−3^) gyres. The higher concentrations were sampled in the equatorial (0.3 mg m^−3^) and the Benguela upwellings (0.22 mg m^−3^), the eastern region of the NATR province (0.22 mg m^−3^) and the eastern section of the North Atlantic (0.22 mg m^−3^), reaching a maximum of 0.45 mg m^−3^ near the Iberian Peninsula (Supplementary Table S1).

### 2.2. Surface *p*PUA Distribution and Biogeographical Patterns

The chemical analyses of the extracts obtained from the large phytoplankton fraction (>10 μm) showed the presence of three polyunsaturated aldehydes: heptadienal (HEPTA), octadienal (OCTA) and decadienal (DECA). Traces of octatrienal were also found in some samples, generally in very low concentrations, near the limit of detection, and ultimately have not been considered.

**Figure 2:**
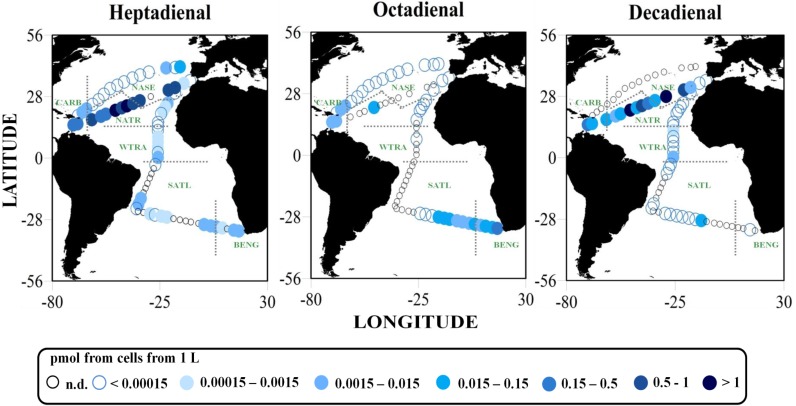
Surface distribution (0–3 m) of potential PUA (*p*PUA) from large-sized phytoplankton (>10 μm). *p*PUA concentrations are shown as pmol from cells in 1 L. Empty small dots denote undetectable *p*PUA concentrations (n.d. in Supplementary Table S1). Increasing color darkness denotes increasing concentrations. See the legend below for detailed ranges. Biogeographical province acronyms are as used in Figure 1. A whisker-box plot of PUA concentrations grouped by bio-geographical ocean region is shown in Supplementary Figure S2.

*p*PUA were present at 95% of the sampling sites (Figure 2; Supplementary Table S1) denoting a widespread distribution of large-sized PUA producers in the surface waters of the open ocean during the sampling period. HEPTA had a wide distribution, being present at 79% of the sampling sites, while OCTA and/or DECA were present at 61% of the sites (Supplementary Table S1). It is known that PUA producers usually release a combination of different aldehydes [6], although less is known about the ratios of production of each species, *i.e.*, the HEPTA:OCTA:DECA ratio, and its variation in relation to environmental conditions. On the basis of the available literature, HEPTA is the most common PUA produced by diatoms [19], in agreement with our observations (Figure 2). However, DECA also reached considerably high concentrations in the transect, T2 (Figure 1; Table 1; Supplementary Table S1) from Cartagena de Indias (Colombia) to Cartagena (Spain). Total *p*PUA (as a summation of OCTA, HEPTA and DECA) showed an average value of 0.22 ± 0.64 pmol from cells in 1 L. The highest values of total *p*PUA, particularly HEPTA and DECA, were found in T2 (Figure 2). The sampling site located in the equatorial upwelling, an area with a high abundance of diatoms, also showed high values of *p*PUA (Figure 2).

**Table 1:** Biogeographical province main characteristics (following Longhurst, 1998). The transects are as in Figure 1. Data are expressed as the average ± SD. PUA, polyunsaturated aldehyde; Chl*a*, chlorophyll *a*.

Province (Acronym)	Date	*N* (Sites/Viable PUA Samples)	Transect	Season	Temperature (°C)	Salinity	Total Chl*a* (mg m^−3^)	Nitrate (μM)	Phosphate (μM)
Caribbean (CARB)	03/23/2011–03/26/2011	4/7	T1	Spring Boreal	25.28 ± 0.62	36.43 ± 0.34	0.05 ± 0.014	0.24 ± 0.22	0.08 ± 0.06
	06/20/2011–06/21/2011	2/2	T2	Summer Boreal	28.95 ± 0.21	35.01 ± 0.01	0.13 ± 0.033	0.45 ± 0.16	0.05 ± 0.02
North Atlantic Subtropical Gyre east (NASE)	12/16/2010–12/24/2010	5/10	T3	Winter Boreal	21.15 ± 1.66	36.82 ± 0.24	0.009 ± 0.060	0.61 ± 0.17	0.04 ± 0.04
	03/26/2011–04/10/2011	12/22	T1	Spring Boreal	17.5 ± 2.83	36.31 ± 0.39	0.250 ± 0.127	1.43 ± 1.50	0.11 ± 0.08
	06/22/2011–06/30/2011	8/8	T2	Summer Boreal	22.47 ± 1.49	37.13 ± 0.35	0.039 ± 0.021	0.40 ± 0.07	0.08 ± 0.01
North Atlantic Tropical Gyral Province (NATR)	12/25/2010–12/30/2011	6/12	T3	Winter Boreal	25.95 ± 1.96	36.5 ± 0.43	0.210 ± 0.046	0.34 ± 0.24	0.04 ± 0.02
	06/30/2011–07/11/2011	9/9	T2	Summer Boreal	27.16 ± 1.40	36.74 ± 0.73	0.078 ± 0.062	0.36 ± 0.12	0.06 ± 0.02
Western Tropical Atlantic (WTRA) (Equatorial upwelling)	12/31/2011–01/10/2012	7/14	T3	Summer Austral	27.9 ± 0.50	35.94 ± 0.67	0.293 ± 0.205	0.35 ± 0.18	0.05 ± 0.06
					27.49	36.10	0.693		
South Atlantic Gyral Province (SATL)	01/19/2011–02/02/2011	23/45	T4	Summer Austral	25.2 ± 2.24	36.43 ± 0.46	0.066 ± 0.028	0.25 ± 0.13	0.10 ± 0.05
Benguela Current Coastal Province (BENG)	02/02/2011–02/05/2011	3/6	T4	Summer Austral	20.96 ± 0.47	35.54 ± 0.16	0.119 ± 0.090	0.32 ± 0.18	0.11 ± 0.03

No significant differences were observed for total *p*PUA concentrations among different provinces (Supplementary Figure S2; Kruskal-Wallis rank sum test, chi-squared = 10.59, *p*-value = 0.059); however, a significant difference was observed when PUAs were analyzed by chain length (Supplementary Figure S2). A Kruskal-Wallis test for HEPTA (chi-squared = 13.30, *p*-value = 0.020) and OCTA (chi-squared = 20.57, *p*-value < 0.001) detected significant differences. Thus, HEPTA was higher in the NATR province (Supplementary Figure S2; Mann–Whitney *post hoc* test; *p* < 0.001), while OCTA was significantly higher in BENG (Mann–Whitney *post hoc* test; *p* < 0.00001), reaching the maximal value measured (0.32 pmol from cells in 1 L).

Significant differences were also found when DECA was compared regionally (Kruskal-Wallis test; chi-squared = 18.48, *p*-value = 0.0024), being more abundant in northern tropical regions (NATR and WTRA) compared with southern ones, but also showed higher values on equatorial upwelling (included in the WTRA province) (Figure 2). OCTA and DECA followed a complementary pattern of distribution regionally (Supplementary Figure S2), with DECA being produced in the tropical Atlantic Ocean. OCTA and DECA distributions showed a significant and negative correlation (Spearman’s rank correlation coefficient = −0.2808), while DECA and HEPTA were positively correlated (Spearman’s rank correlation coefficient = 0.4569).

The latitudinal northern transects (T1 and T2 in Figure 1) included the provinces, CARB, NASE and NATR. Within these areas, plankton is subject to considerable seasonal variation in the environmental forcing [23,25]. Blooms of large diatoms usually develop in mid-April (>5 μm), and a transition to oligotrophic conditions occurs at the end of June. Transect T1 was sampled during March–April (boreal spring), and transect T2 was sampled during June–July (boreal summer); thereby, seasonal differences in the abundance and composition of phytoplanktonic community must be expected.

Phytoplankton biomass along T2 (averaged TChl*a*: 0.07 ± 0.05 mg m^−3^) was much lower than that obtained for T1 (averaged TChl*a*: 0.19 ± 0.14 mg m^−3^), and significant differences in nutrient availability were observed between western and eastern *Sargassum* (Figure 2, Table 1). The summer population of NASE (0.039 ± 0.021 mg Chl*a* m^−3^) was associated with highly oligotrophic conditions (Table 1) and produced significantly higher amounts of PUA compared to spring or winter communities (Supplementary Figure S3) (Kruskal-Wallis test, chi-squared = 10.0766, *p*-value = 0.006485). When we consider each PUA chain length separately, DECA and HEPTA were higher in summer, compared to OCTA*,* which was not detected (Supplementary Table S1).

Our results show that NASE assemblages collected in summer showed higher *p*PUA production and a different PUA production ratio than that collected in spring or winter, with an apparent increase of DECA production. Moreover, as occurred for the comparison of different biogeographical regions (Supplementary Figure S2), DECA and OCTA production followed a complementary temporal pattern, with higher DECA in summer associated with lower OCTA (Supplementary Figure S3).

The production of PUA has been shown to be strain dependent [26]. Different strains of the same species, isolated in different seasons, produced different quantities of PUA in culture. Taylor and collaborators demonstrated that summer strains of *Skeletonema marinoi* had a higher production of PUA [26] and postulated that strains that were isolated during times of higher grazing pressure and less beneficial nutrient conditions had higher *p*PUA production. This is consistent with our results, thus extending to natural assemblages under physiological (nutrient) stress. This fact is especially interesting, because the observed patterns of variability of *p*PUA in nature shows the net effect of taxonomical, physiological and environmental effects, which are hard to reproduce in laboratory conditions.

Seasonal environmental changes, such as day length or temperature, could also impact PUA persistence in water, toxicity or the signaling role. It has been experimentally demonstrated that temperature significantly affects the persistence of HEPTA, OCTA and DECA in natural Atlantic seawater, being more persistent at 10 °C than at either 15 °C or 20 °C. Moreover, DECA was consistently more persistent than OCTA and HEPTA at the higher temperature assayed [27]. The higher persistence of DECA at warmer waters compared with HEPTA or OCTA could imply less sensitivity to DECA of the low latitude strains (and species) compared with the native species of higher latitudes. Depending on the average levels of exposure of the cells to released PUAs, the type and quantity of these compounds could have a different effect on plankton community structure in temperate waters compared with, for instance, polar and sub-polar environments.

Our main interest in this work was not to identify PUA producers, but to describe the range and composition of PUAs potentially present in open oceanic areas. In fact, we should not rule out the possibility that other non-diatom large-sized class producers were responsible for the *p*PUA concentration observed. Most oligotrophic oceanic areas are dominated by picoplanktonic species [28], which would not be collected by a big-sized mesh, such as the one used in our sampling. However, large-sized colonies (>1 mm) of *Trichodesmium* (Cyanophyta) are especially abundant in oligotrophic tropical regions characterized by warm (>22° C) surface waters [25], the equatorial Atlantic region from 5°S to 15°N and also are present in the North Atlantic Subtropical Gyre and the western Sargasso Sea [28,29,30]. There are no reports regarding PUA production from Cyanophyta; however, *Trichodesmium* is able to synthesize polyunsaturated fatty acids (PUFA), such as eicosapentaenoic acid (EPA) and arachidonic acid (AA) [31]. These compounds may act as HEPTA and DECA precursors, respectively [5,6]. Other non-diatom DECA producers, such as the colony-forming *Phaeocystis pouchetii* (Prymnesiophyte), may also produce DECA [32]. Therefore, *p*PUA should not be strictly associated to diatoms, as other producers as *Trichodesmium* trichomes or *Phaeocystis* colonies are plausible.

2.3. *p*PUA Distribution and Trophic Gradients

**Figure 3:**
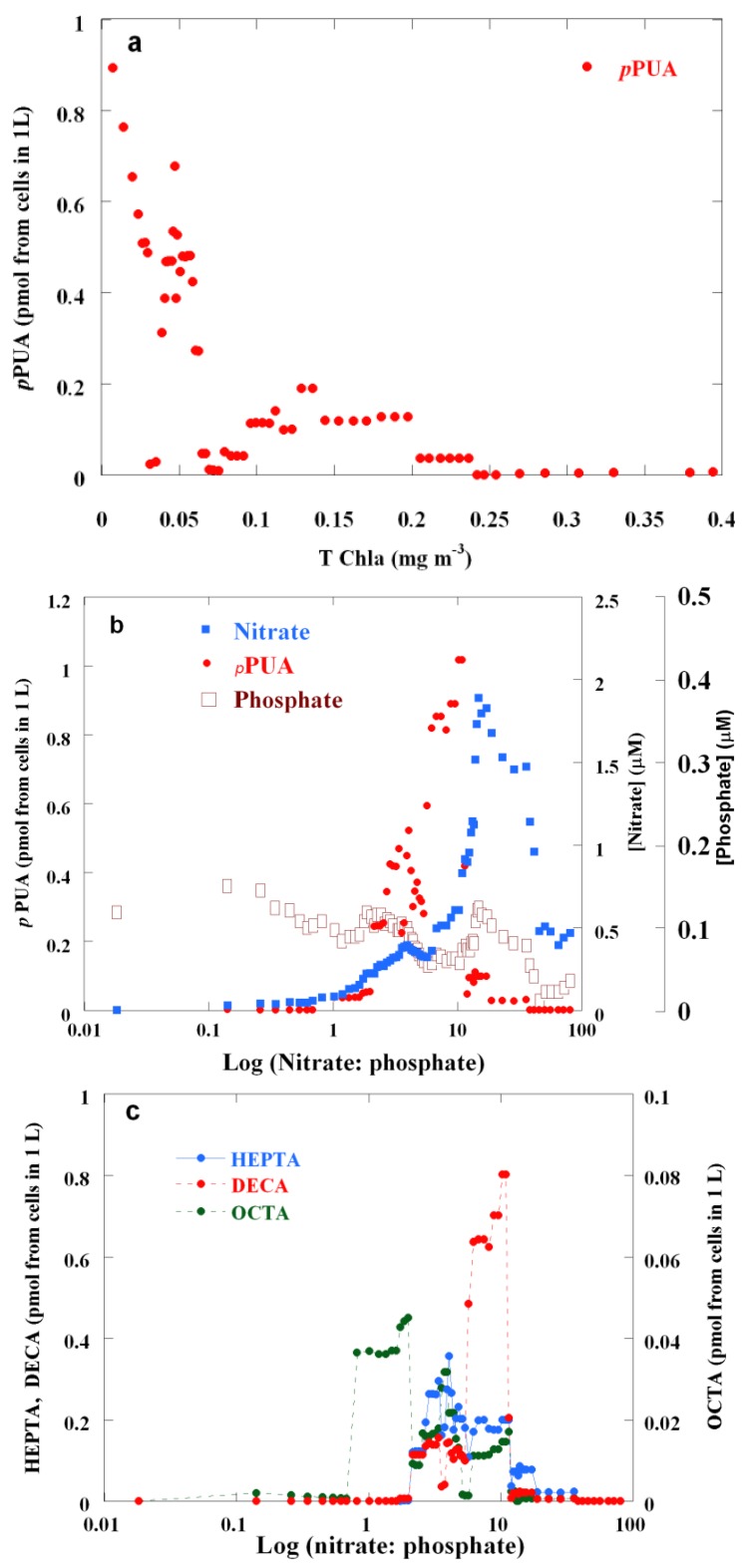
Nine data running averages of (**a**) *p*PUA over a gradient of total chlorophyll *a* (TChl*a*), (**b**) *p*PUA and nutrients (nitrate and phosphate) over the gradient of the nitrate to phosphate ratio and (**c**) different chain lengths of aldehydes, heptadienal, octadienal and decadienal, over the gradient of the nitrate to phosphate ratio.

The biogeographical regions described above embrace a wide range of ecosystems with diverse productivity, such as oligotrophic subtropical gyres, upwellings and THE mesotrophic shelf system. Such trophic gradation is evident in total chlorophyll and nutrient distributions (Figure 1, Supplementary Table S1). From ultraoligotrophic (TChl*a* < 0.06 mg m^−3^, *sensu* [33]) to mesotrophic conditions (TChl*a* 0.1–0.3 mg m^−3^, *sensu* [33]), large phytoplankton showed decreasing *p*PUA levels (Figure 3a), suggesting trophic control of PUA production. Examination of the relationship between *p*PUA and the availability of dissolved inorganic nutrients (nitrogen and phosphorus ratio, N:P) showed that the lowest (N:P < 1) and the highest (N:P > 20) available N:P were coincident with the lowest *p*PUA concentrations (Figure 3b). N:P ratios from two to 15 were related to the highest *p*PUA concentrations.

PUA production is dependent on taxonomic composition, cellular PUFA contents (PUA precursors) and enzymatic lipoxygenase activity. These variables are directly related to the cell physiological state and the trophic state of the surrounding environment and could explain the observed trend. Our results strongly suggest the control of nutrient availability on the regulation of *p*PUA production in large-sized phytoplankton assemblages of the Atlantic Ocean. This nutrient control has been previously observed by other authors in experimental conditions in mesocosms [34]. Several researchers showed that a single species can significantly change the type and the quantity of PUAs, depending on nutrient availability [35,36]. Ribalet and coworkers [34] proposed that the *p*PUA production of the diatom, *Skeletonema marinoi* (mainly heptadienal and octadienal), was limited by the amount of substrates (PUFA, polyunsaturated fatty acids) under P-limitation and by enzyme activity under N-limitation. This hypothesis could explain the trends represented in Figure 3, but the goal here is that the present results have been obtained for natural assemblages of mixed species of cells probably subjected to a chronic nutrient limitation.

Octadienal, but especially heptadienal and decadienal, showed an alternance in dominance along the N:P gradient (Figure 3c), and their distributions were negatively correlated (Spearman correlation coefficient: −0.280869), suggesting qualitative changes in the biochemical routes of PUA production in relation to nutrients variability. Recently, it has been described that in low-phosphorus marine systems, phytoplankton cells replace membrane phospholipids with non-phosphorus lipids [37]. Likewise, the ratios of PUAs that are produced could be affected by the differences in the biosynthetic pathways of the various PUA precursors. OCTA is mainly biosynthesized by glycolipids routes; DECA is mainly biosynthesized by phospholipids routes, and HEPTA can be synthesized by both glycolipids and phospholipids routes [5,38]. Hence, membrane remodeling under nutrient stress would affect the pool of PUA precursors, varying the *p*PUA ratios of the phytoplankton population.

The *p*PUA concentrations obtained from a phytoplankton fraction >1.2 μm in a bloom of *Skeletonema marinoi* in Adriatic Sea ranged from 18 to 25 nmol from cells in 1 L ([18]; *p*PUA was referred to in this publication as particulate PUA). However, *p*PUA measured in the Atlantic Ocean did not reach concentrations of the nanomolar scale, differing three orders of magnitude from that found in the diatom bloom in the Adriatic Sea. Some possible reasons may be proposed for explaining such differences. First, the phytoplankton size fractions considered are significantly different in the two studies. *p*PUA in the coastal bloom of *S. marinoi* was related to the phytoplankton fraction larger than 1.2 μm, while our study was addressed on the size fraction larger than 10 μm. Second, cell densities during bloom events could reach 10^6^ cell L^−1^, which represents three to four orders of magnitude higher cell densities than that usually measured in open ocean areas (10^2^–10^3^ cell L^−1^). Due to the low cell densities typically found in the open ocean, the *p*PUA concentrations measured for the large-sized phytoplankton fraction in open oceanic areas seem reasonable. Moreover, marine diatoms can span from a few micrometers to millimeters in length [39], and probably, lower-sized diatoms would also contribute significantly to the released PUA pool in the ocean. 

**Figure 4:**
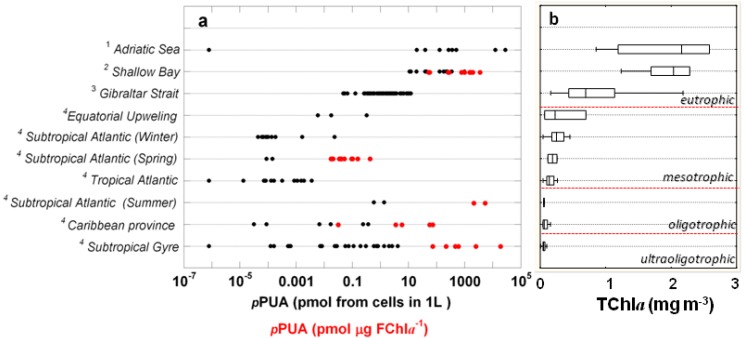
(**a**) *p*PUA expressed as picomoles (pmol) from cells in 1 L (black spots) and *p*PUA normalized by FChl*a* > 10 μm (pmol μg FChl*a*^−1^) (red spots) concentrations measured at various oceanic areas (see the left axis). Data were grouped following the trophic gradient based on total chlorophyll concentration (see the sources below). (**b**) Box-plot for the ranges of total chlorophyll (TChl*a*) in ecological areas. Ocean areas were arranged on the vertical axes in relation to mean Chl*a*. *Sources*: ^1^ *p*PUA for phytoplankton > 1.2 μm (*n* = 8, [18]); total chlorophyll was derived from remotely sensedmeasurements ([42]). ^2^ *p*PUA for phytoplankton > 10 μm; total Chl*a* was measured in the field through standard fluorometric methods (*n* = 12, [43]). ^3^ *p*PUA for phytoplankton > 10 μm; total Chl*a* was measured in the field through standard fluorometric methods (*n* = 68; [44]). ^4^ This work.

We extended the trophic gradient of this study by including the published and unpublished data of phytoplankton samples collected in marine coastal areas (Figure 4). *p*PUA concentrations ranged from pM (equatorial upwelling) to micromole (Adriatic Sea and Shallow Bay) concentrations. The highest *p*PUA concentrations were measured in coastal areas with TChl*a* over 1 mg m^−3^ and the lowest concentrations were found in subtropical and tropical Atlantic waters with TChl*a* around 0.2 mg m^−3^. This result can be explained from the increasing abundance of PUA producers towards eutrophic conditions. In contrast, *p*PUA was higher under ultra-oligotrophic conditions (TChl*a* < 0.1 mg m^−3^, *sensu* [33]). Since the fraction of large phytoplankton expressed as TChl*a* is especially low at ultraoligotrophic conditions [40], the high *p*PUA values under oligotrophic conditions are even more evident when *p*PUAs are normalized by large-sized phytoplankton biomass when available (expressed as pmol PUA μg Chl*a*^−1^) (Figure 4). We relate this result to the nutrient stress of *p*PUA producers, in agreement with Graneli *et al.* [41]. They hypothesized an increase in the release rate of chemical compounds associated with N:P unbalanced phytoplankton communities.

In this work, we report, for the first time, data on the *p*PUA distribution for open oceanic areas. The concentration ranges derived from large phytoplankton are related to trophic conditions. However, the sources of variability of *p*PUA are diverse, and data on natural ambient ones are still scarce. Future studies centered on the seasonal variation of *p*PUA and the depth profiles of dissolved PUA would be a useful next step toward understanding the ecological relevance of these compounds in nature. Physiological variables, such as cell lysis rates, should be also related to natural dissolved concentrations of PUA. Since PUA production prevails under certain trophic conditions and among certain taxa (e.g., large eukaryotes, such as diatoms), PUAs could also take part in determining the composition and size structure of the phytoplankton community.

## 3. Experimental Section

### 3.1. Study Area

Samples for polyunsaturated aldehyde extractions were collected in several cruises carried out on board the B/O Hespérides and B/O Sarmiento de Gamboa from December 2010 to July 2011 (Supplementary Table S1). A total of 71 sites were visited, and 137 viable surface samples of the phytoplankton fraction > 10 μm were collected at four different transects (T1 to T4 in Figure 1) Temperature, salinity and chlorophyll fluorescence were monitored using a CTD (SeaBird 9 *plus*, Bellevue, WA, USA). 

### 3.2. Sampling

Water samples were collected by means of 12-L Niskin bottles mounted on a rosette frame. A volume of 250–500 mL was collected for the total and fractionated (>10 μm) Chl*a* determination, filtered through Whatman^®^ glass microfiber filters (25 mm diameter, grade GF/F, Sigma-Aldrich, Buchs, Switzerland) and kept frozen for at least 6 hours until extraction. Pigments were extracted by placing them in 5–7 mL of 90% acetone at 4°C for 24 h, and the fluorescence of the extract was determined by means of a Turner Designs fluorometer [45] calibrated with a Chl*a* standard (Sigma-Aldrich, Buchs, Switzerland). No phaeophytin correction was applied. Total chlorophyll (TChl*a*) was measured in all sampling sites, but fractionated chlorophyll was only measured at T1 and T2.

Samples used for dissolved nutrients analyses were drawn from the Niskin bottles into polyethylene vials and immediately frozen (−20 °C) until analysis. Concentrations were measured spectrophotometrically with a Skalar autoanalyzer following standard procedures [46,47]. The detection limits of concentrations were 0.01 and 0.02 μM for NO_3_ + NO_2_ and PO_4_, respectively.

At each sampling site, two samples of a variable, but controlled, volume (from 50 to 450 L) of surface seawater (3 m) were collected for *p*PUA quantification. Natural seawater was pumped at 11–12 L min^−1^. The sample volume was selected in relation to the CTD fluorescence signal. We generally collected a high water volume, considering that the open ocean is oligotrophic (TChl*a* < 0.1 mg m^−3^). To quantify *p*PUA in large phytoplankton, water samples were firstly passed through a 200-μm mesh to eliminate the zooplankton fraction and then through a 10-μm mesh. Phytoplankton cells retained on the 10 μm mesh were concentrated into 100 mL vials by using 0.2 μm filtered seawater. Concentrated samples were filtered at low vacuum pressure onto polycarbonate filters (Nucleopore, 0.4 μm). The phytoplankton cells retained were rinsed from the filter with 1 mL of 25 mM of *O*-(2,3,4,5,6-pentafluorobenzyl) hydroxylamine hydrochloride (PFBHA, Fluka, Basel, Switzerland) solution in 100 mM Tris HCl, pH 7.2 [48], kept refrigerated or immersed in liquid nitrogen, depending on the cruise length, and transported to the lab. Once at the lab, samples were conserved at −80 °C until extraction.

### 3.3. Polyunsaturated Aldehydes Extraction and Quantification

Each sample was unthawed and immediately transferred to a conical glass vial, and 5 μL of internal standard were added (benzaldehyde, 1 mM in methanol, Sigma-Aldrich, Buchs, Switzerland). Samples were artificially disrupted by sonication on ice following the recommendations of Wichard *et al.* [48], and then, the samples were kept 1 h at ambient temperature to ensure a complete derivatization of aldehydes. For extraction, 0.5 mL of methanol and 1 mL of hexane were added, and the sample was vortexed for 1 min. The mixture was acidified by the addition of sulfuric acid and vortexed again [18]. The hexane phase was taken to dryness under vacuum and kept at −80 °C until analysis by gas chromatography-mass spectrometry (GC-MS). Before GC-MS analysis, the samples were dissolved in 50 μL of hexane and transferred into a 100-μL glass micro insert (Waters^®^ VWR International™, Radnor, PA, USA) closed with caps fitted with a PTFE septum.

GC-MS analysis was performed using an Agilent 7890A GC (Agilent Technologies Inc., Santa Clara, CA, USA) coupled to a Waters Micromass Quattro Micro (Milford, MA, USA) and equipped with a 30-m DB-5MS column (0.25 mm internal diameter, 0.25 μm stationary phase thickness, 95% dimethylpolysiloxane, 5% diphenylpolysiloxane). The GC oven temperature was programmed as follows: held at 60 °C for 1 min, then increased at 5 °C per minute to 300 °C and finally held at 300 °C for 1 min. The inlet temperature was 250 °C in splitless mode (1 μL). Helium was the carrier gas at a constant flow of 1 mL/min. The electron energy was 70 eV. The PUA-PFBHA derivatives were identified by comparison to external standards and the presence of their molecular ions (*m/z* 305 for heptadienal, 319 for octadienal and 347 for decadienal) and also the presence of major fragments ions, *i.e.*, *m/z* 276 for the PUAs, 271 for the internal standard and 181 for all. Quantification was based on the ratio between the fragment at *m/z* 276 of the derivatized PUA and *m/z* 271 of the derivatized internal standard (that is, benzaldehyde). The calibration was performed using a synthetic standard of derivatized (25 mM PFBHA-solution in Tris-HCl at pH 7.2) 2*E*,4*E*-heptadienal (90%, Sigma-Aldrich Chemie GmbH, Steinheim, Germany), 2*E*,4*E*-octadienal (≥96%, Sigma-Aldrich Chemie GmbH) and 2*E*,4*E*-decadienal (85%, Sigma-Aldrich Chemie GmbH) in ranges from 0.5–15 nM, 15–200 nM and 200–8000 nM, to cover the wide range of molarities found in the concentrated extracts of the samples (a maximum value of 4200 nM). Calibration curves were repeated with every group of analyzed samples for three different ranges: 0.5–15 nM, 15–200 nM and 200–8000 nM of each aldehyde. The correlation coefficients obtained for each aldehyde at every range were: *R*^2^ = 0.9884 ± 0.009, 0.9919 ± 0.011 and 0.9963 ± 0.004 for heptadienal; *R*^2^ = 0.9875 ± 0.013, 0.9716 ± 0.037 and 0.9896 ± 0.011 for octadienal; *R*^2^ = 0.9509 ± 0.046, 0.9823 ± 0.005 and 0.9915 ± 0.006 for decadienal, respectively.

The peak area ratios of each aldehyde (*m/z* 276), relative to the internal standard (*m/z* 271), was plotted against the concentration of the aldehyde. The obtained concentrations were finally normalized to the filtered volume of seawater, and the results were expressed as the molarity of PUA obtained from cells contained in 1 L of seawater.

### 3.4. Statistics

Statistical analysis of the data was performed by using statistical package R. All data were checked for normality and homoscedasticity before analysis by using the Shapiro-Wilk test (*p*-value > 0.05) and Levene’s test (*p*-value > 0.05), respectively. The Kruskal-Wallis rank sum test and *post hoc* pairwise comparison by means of the Mann–Whitney test were applied for comparing *p*PUA levels.

## 4. Conclusions

In the present study, we provided the first assessment of *p*PUA concentrations in the open ocean and demonstrate that large-sized PUA producers are widely spread in the Atlantic Ocean. Heptadienal was the most widely distributed one. Other *p*PUAs, such as octadienal or decadienal, also dominated, depending on the site or season. Our results showed a relationship between *p*PUA in large-sized phytoplankton and nutrient availability, with higher *p*PUA concentrations under ultraoligotrophic conditions and at intermediate nitrate:phosphate ratios (from two to 10). Given that Chl:biomass ratio also varies in relation to the physiological status of phytoplankton, the PUA concentration in future studies should be related to conservative biomass variables, such as carbon instead of chlorophyll. Finally, we have found that the quantity of these compounds can range from the pM to μM level in the ocean, from ultraoligotrophic to eutrophic conditions.

## Abbreviations

NASA: National Aeronautics and Space Administration
MODIS: Moderate Resolution Imaging Spectroradiometer
GSFC: Goddard Space Flight Center
CTD: Conductivity/Temperature/Depth profiler
GC-MS: Gas Chromatography-Mass spectrometry
PTFE: Polytetrafluoroethylene
INDIAL: Acronym for the spanish project “Regulación Ambiental e Implicaciones tróficas de la liberación de aldehídos volátiles por diatomeas”
CEI·MAR: Campus of International Excellence of the Sea

## Supplementary Files

Supplementary File 1 Supplementary Information (PDF, 433 KB)
