# Supplementary material for: Polyunsaturated Aldehydes from Large Phytoplankton of the Atlantic Ocean Surface (42°N to 33°S)
Source: Mar Drugs. 2014 Jan 27;12(2):682–99. doi: 10.3390/md12020682 (PMC3944509; doi:10.3390/md12020682)
Supplement: Supplementary File 1 — Supplementary Information (PDF, 433 KB) [file marinedrugs-12-00682-s001.pdf]

Supplementary Information

Figure S1. Latitudinal variation of Sea Surface Temperature (SST) during the cruises. T: Transects as in Figure 1.

Figure S2. Box plot of (**a**) *p*PUA (*p*HEPTA + *p*OCTA + *p*DECA), (**b**) HEPTA, (**c**) OCTA, and (**d**) DECA, grouped by biogeographical region following Longhurst criteria (See Table S1 for detailed data set). Line represent median. Longhurst biogeographical provinces as in Figure 1.

Figure S3. Box plot of (**a**) *p*PUA, (**b**) *p*HEPTA, (**c**) *p*OCTA, and (**d**) *p*DECA, in stations sited in North Atlantic Subtropical grouped by season (winter, spring and summer) (See Table S1 for main characteristics). Bars represent 25% to 75% of observed data. Line represent median. Dark circles: extreme data. Note that Y axis is at logarithmic scale.

Figure S4. *p*PUA expressed as pmol μgFChla^{-1} (FChl*a* > 10 μm) obtained along Transect 1 (T1 in Figure 1) and Transect 2 (T2 in Figure 1).

Table S1. Detailed data set obtained for all stations sampled. **Trans**: Transect, T1 to T4 as in Figure 1; **Prov**: Biogeographical province following Longhurst criteria [23]; **Lat**: Latitude (°); **Long**: Longitude (°); **T**: Temperature (°C); **S**: Salinity; **Vol**: Seawater volume (L); **HEPTA**: Heptadienal (pmol from cells in 1 L); **OCTA**: Octadienal (pmol from cells in 1 L); **DECA**: Decadienal (pmol from cells in 1 L); **TPUA**: Total PUA (as summation of HEPTA + OCTA + DECA); **TChla**: Total Chlorophyll a (mg m^{-3}): **FChla**: Fractionated chlorophyll, from larger size phytoplankton fraction (>10 μm) (mg m^{-3}); **Nit**: Nitrate (μM); **Pho**: Phosphate (μM). When replicates data are expressed as average ± SD. **n.d.**: Non detectable levels. "**-**": No samples.

Figure S1. Latitudinal variation of Sea Surface Temperature (SST) during the cruises. T: Transects as in Figure 1.

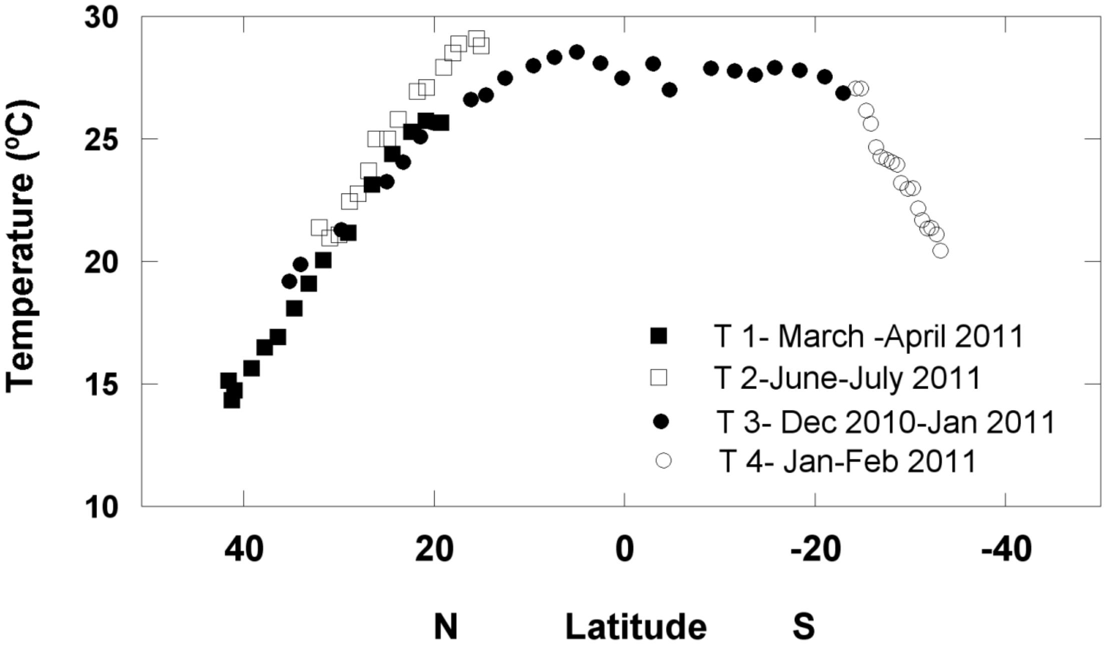

Figure S2. Box plot of (**a**) *p*PUA (*p*HEPTA + *p*OCTA + *p*DECA), (**b**) HEPTA, (**c**) OCTA, and (**d**) DECA, grouped by biogeographical region following Longhurst criteria (See Table S1 for detailed data set). Line represent median. Longhurst biogeographical provinces as in Figure 1.

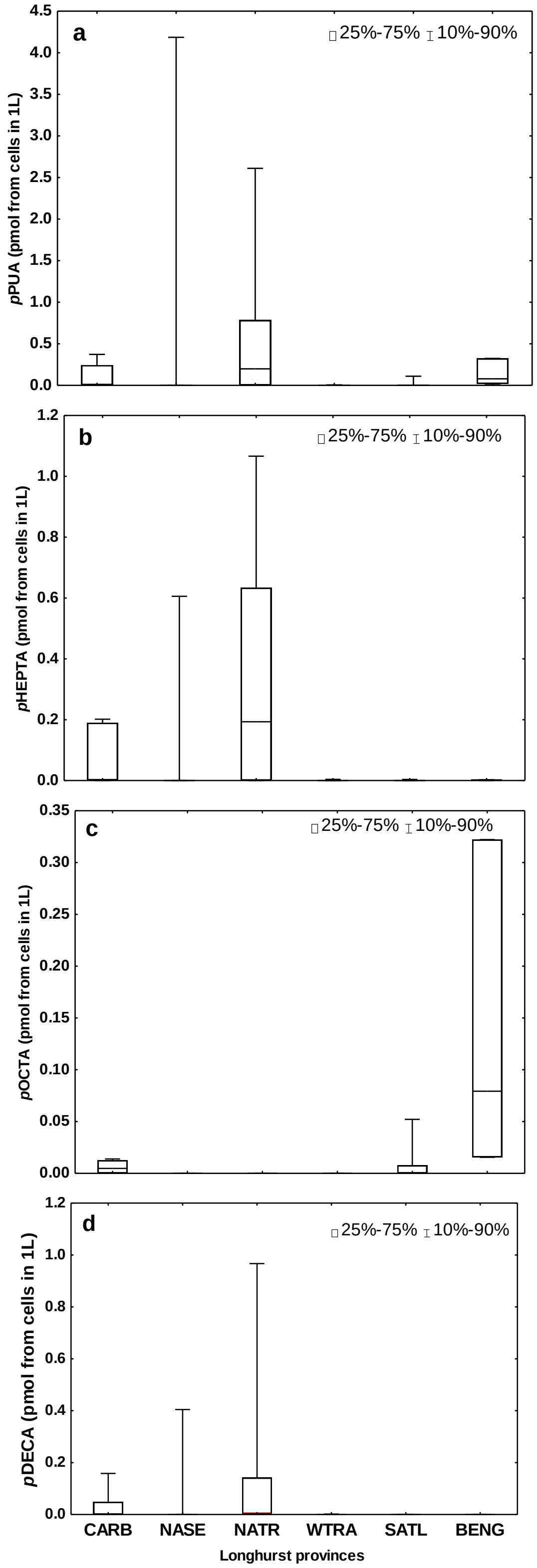

Note that scales are different.

Figure S3. Box plot of (**a**) *p*PUA, (**b**) *p*HEPTA, (**c**) *p*OCTA, and (**d**) *p*DECA, in stations sited in North Atlantic Subtropical grouped by season (winter, spring and summer) (See Table S1 for main characteristics). Bars represent 25% to 75% of observed data. Line represent median. Dark circles: extreme data. Note that Y axis is at logarithmic scale.

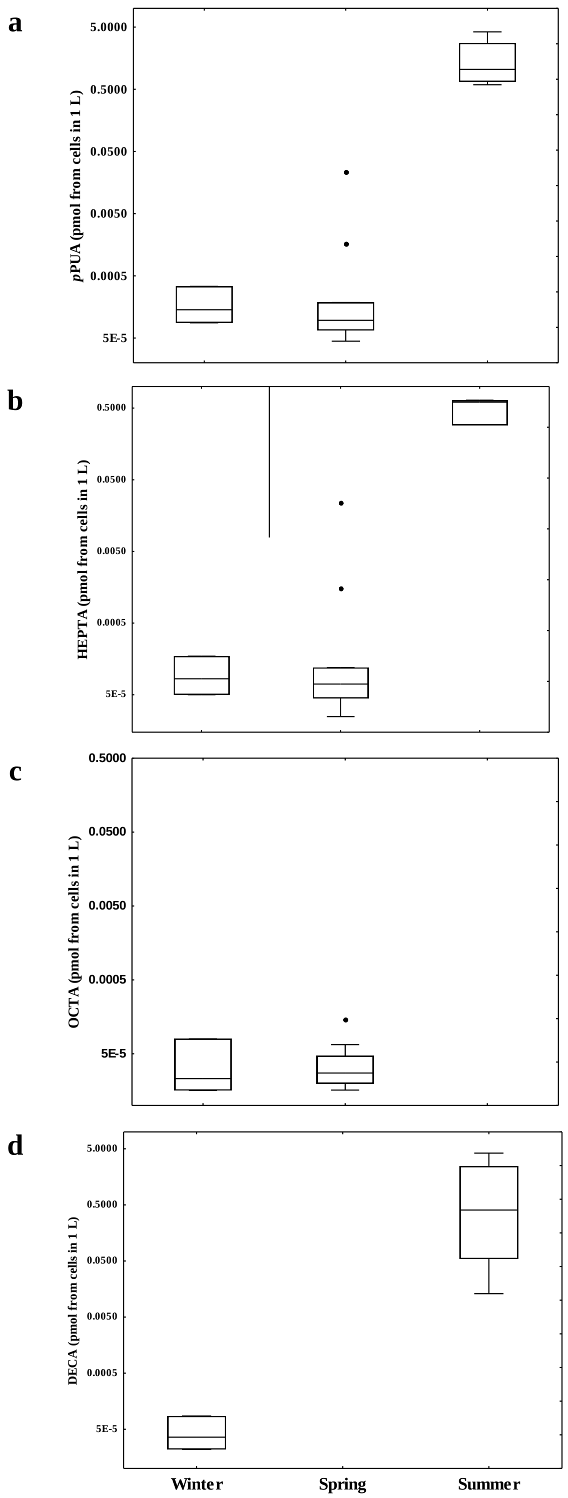

Figure S4. *p*PUA expressed as pmol μgFChla^{-1} (FChl*a* > 10 μm) obtained along Transect 1 (T1 in Figure 1) and Transect 2 (T2 in Figure 1).

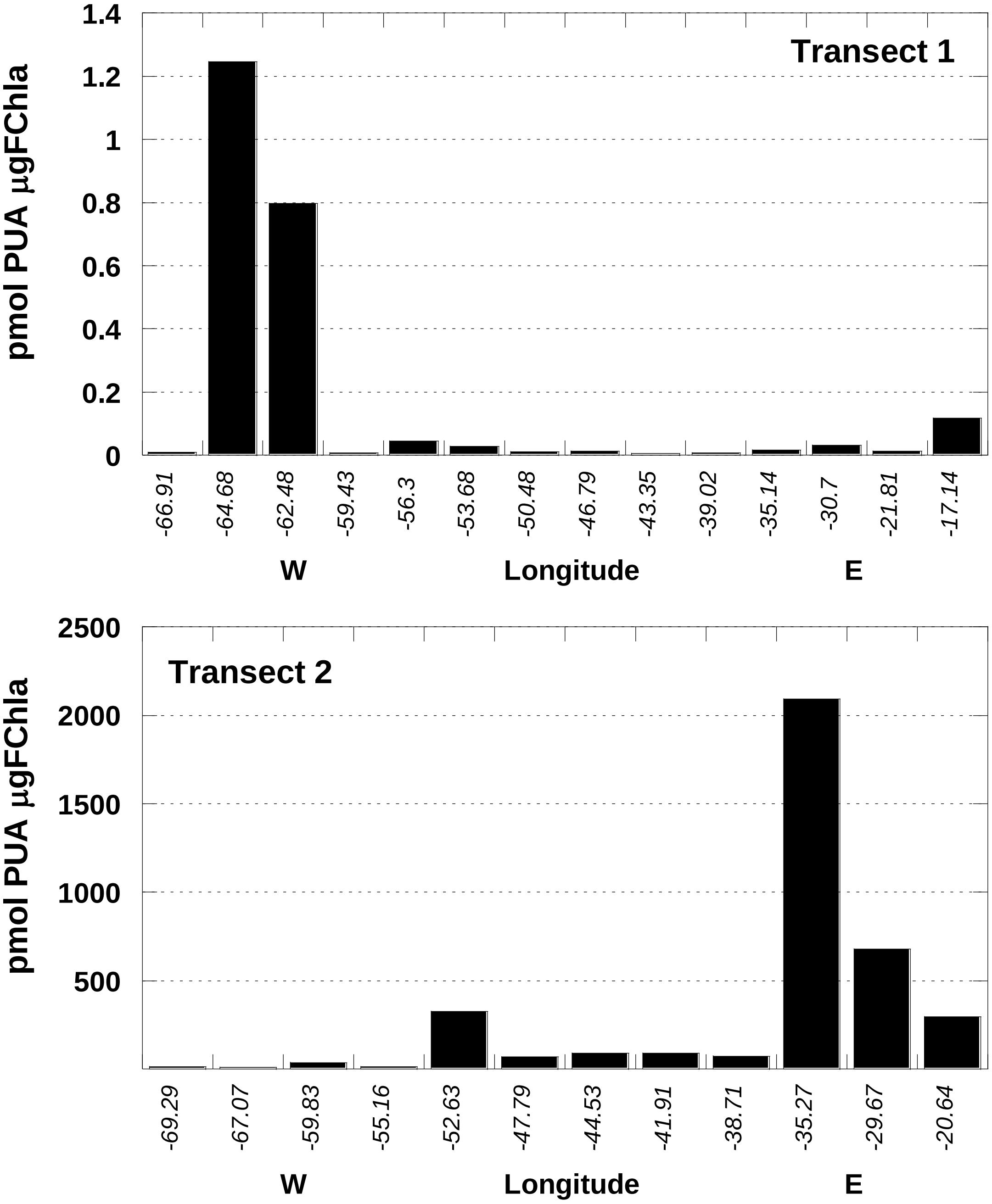

Table S1. Detailed data set obtained for all stations sampled. **Trans**: Transect, T1 to T4 as in Figure 1; **Prov**: Biogeographical province following Longhurst criteria [23]; **Lat**: Latitude (°); **Long**: Longitude (°); **T**: Temperature (°C); **S**: Salinity; **Vol**: Seawater volume (L); **HEPTA**: Heptadienal (pmol from cells in 1 L); **OCTA**: Octadienal (pmol from cells in 1 L); **DECA**: Decadienal (pmol from cells in 1 L); **TPUA**: Total PUA (as summation of HEPTA + OCTA + DECA); **TChla**: Total Chlorophyll a (mg m^{-3}): **FChla**: Fractionated chlorophyll, from larger size phytoplankton fraction (>10 μm) (mg m^{-3}); **Nit**: Nitrate (μM); **Pho**: Phosphate (μM). When replicates data are expressed as average ± SD. **n.d.**: Non detectable levels. "**-**": No samples.

Date	Trans	Prov	Lat	Long	T	S	Vol	HEPTA	OCTA	DECA	TPUA	TChla	FChla	Nit	Pho
24/03/11	T1	CARB	19.26	−66.91	25.68	36.02	100	$5.9 \times 10^{-5} \pm 8.6 \times 10^{-6}$	$3.0 \times 10^{-5} \pm 1.9 \times 10^{-8}$	n.d.	$8.8 \times 10^{-5} \pm 8.6 \times 10^{-6}$	0.07	0.01	-	-
25/03/11	T1	CARB	20.88	−64.68	25.75	-	100	$3.3 \times 10^{-3} \pm 4.4 \times 10^{-3}$	$1.4 \times 10^{-2} \pm 1.9 \times 10^{-2}$	n.d.	$1.7 \times 10^{-2} \pm 2.3 \times 10^{-2}$	0.05	0.01	0.48	0.04
26/03/11	T1	CARB	22.41	−62.48	25.30	36.69	100	3.1×10^{-3}	3.7×10^{-3}	n.d.	6.8×10^{-3}	0.04	0.01	0.19	0.16
27/03/11	T1	CARB	24.43	−59.43	24.41	36.73	100	$2.3 \times 10^{-5} \pm 5.4 \times 10^{-6}$	$7.8 \times 10^{-6} \pm 5.7 \times 10^{-7}$	n.d.	$3.1 \times 10^{-5} \pm 4.8 \times 10^{-6}$	0.04	0.00	0.04	0.04
28/03/11	T1	NASE	26.53	−56.30	23.14	36.98	100	$2.5 \times 10^{-5} \pm 1.4 \times 10^{-5}$	$2.0 \times 10^{-5} \pm 1.5 \times 10^{-5}$	n.d.	$4.5 \times 10^{-5} \pm 3.0 \times 10^{-5}$	0.04	0.00	0.05	0.04
29/03/11	T1	NASE	29.01	−53.68	21.17	36.88	100	$3.5 \times 10^{-5} \pm 1.9 \times 10^{-5}$	$2.6 \times 10^{-5} \pm 1.7 \times 10^{-5}$	n.d.	$6.1 \times 10^{-5} \pm 3.6 \times 10^{-5}$	0.05	0.00	0.03	0.04
30/03/11	T1	NASE	31.62	−50.48	20.07	36.69	100	$4.5 \times 10^{-5} \pm 4.2 \times 10^{-6}$	$2.2 \times 10^{-5} \pm 5.9 \times 10^{-6}$	n.d.	$6.6 \times 10^{-5} \pm 1.0 \times 10^{-5}$	-	-	0.05	0.03
31/03/11	T1	NASE	33.13	−46.79	19.10	36.59	100	$5.8 \times 10^{-5} \pm 2.5 \times 10^{-5}$	$2.7 \times 10^{-5} \pm 2.4 \times 10^{-5}$	n.d.	$8.6 \times 10^{-5} \pm 5.0 \times 10^{-5}$	0.26	0.01	0.15	0.03
01/04/11	T1	NASE	34.68	−43.35	18.09	36.46	100	$4.9 \times 10^{-5} \pm 1.8 \times 10^{-5}$	$1.9 \times 10^{-5} \pm 8.1 \times 10^{-6}$	n.d.	$6.8 \times 10^{-5} \pm 2.6 \times 10^{-5}$	0.23	0.01	0.77	0.06
02/04/11	T1	NASE	36.43	−39.02	16.93	36.25	100	$7.0 \times 10^{-5} \pm 2.1 \times 10^{-5}$	$3.1 \times 10^{-5} \pm 2.5 \times 10^{-5}$	n.d.	$1.0 \times 10^{-4} \pm 4.5 \times 10^{-5}$	0.30	0.02	3.85	0.22
03/04/11	T1	NASE	37.79	−35.14	16.48	36.18	100	$8.0 \times 10^{-5} \pm 2.2 \times 10^{-5}$	$1.6 \times 10^{-5} \pm 8.9 \times 10^{-6}$	n.d.	$9.6 \times 10^{-5} \pm 3.1 \times 10^{-5}$	0.36	0.01	1.18	0.09
04/04/11	T1	NASE	39.13	−30.70	15.63	36.02	100	$9.0 \times 10^{-5} \pm 1.3 \times 10^{-5}$	$4.6 \times 10^{-5} \pm 8.5 \times 10^{-6}$	n.d.	$1.4 \times 10^{-4} \pm 2.2 \times 10^{-5}$	0.25	0.01	1.89	0.13
06/04/11	T1	NASE	41.01	−21.81	14.74	35.94	100	$1.5 \times 10^{-3} \pm 1.5 \times 10^{-3}$	$1.4 \times 10^{-4} \pm 6.8 \times 10^{-5}$	n.d.	$1.6 \times 10^{-3} \pm 1.6 \times 10^{-3}$	0.38	0.05	2.41	0.15
07/04/11	T1	NASE	41.24	−17.14	14.34	35.86	100	$1.2 \times 10^{-4} \pm 1.3 \times 10^{-5}$	$6.7 \times 10^{-5} \pm 4.7 \times 10^{-5}$	n.d.	$1.9 \times 10^{-4} \pm 6.0 \times 10^{-5}$	0.18	0.01	4.15	0.28
08/04/11	T1	NASE	41.57	−14.73	15.14	35.99	100	$2.3 \times 10^{-2} \pm 3.3 \times 10^{-2}$	$4.7 \times 10^{-5} \pm 6.6 \times 10^{-5}$	n.d.	$2.3 \times 10^{-2} \pm 3.3 \times 10^{-2}$	0.45	0.20	0.17	0.06
22/06/11	T2	CARB	15.07	−69.29	28.80	35.51	184	2.0×10^{-1}	1.3×10^{-2}	1.6×10^{-1}	3.7×10^{-1}	0.11	0.03	0.34	0.07
23/06/11	T2	CARB	15.58	−67.07	29.10	35.52	148	1.9×10^{-1}	6.0×10^{-3}	4.9×10^{-2}	2.4×10^{-1}	0.16	0.02	0.57	0.04
25/06/11	T2	NATR	17.43	−59.83	28.89	35.54	74.9	5.0×10^{-1}	n.d.	1.4×10^{-1}	6.4×10^{-1}	0.13	0.02	0.34	0.03
27/06/11	T2	NATR	19.02	−55.16	27.92	36.60	85.7	1.9×10^{-1}	n.d.	5.4×10^{-3}	2.0×10^{-1}	0.01	0.01	0.45	0.09
28/06/11	T2	NATR	20.01	−52.63		36.74	55.21	2.6×10^{-1}	n.d.	5.4×10^{-2}	3.1×10^{-1}	0.06	0.02	0.51	0.05
30/06/11	T2	NATR	21.74	−47.79	26.94	37.09	45.3	1.1	8.8×10^{-2}	1.4	2.6	0.05	0.01	0.40	0.05

Table S1. *Cont.*

01/07/11	T2	NATR	22.86	−44.53		37.05	44.7	7.7×10^{-1}	n.d.	1.6×10^{-2}	7.8×10^{-1}	0.05	0.01	-	-
02/07/11	T2	NATR	23.73	−41.91	25.80	37.45	23	1.1	n.d.	9.7×10^{-1}	2.0	0.06	0.02	0.17	0.07
03/07/11	T2	NATR	24.86	−38.71	25.01	37.56	56	6.3×10^{-1}	n.d.	1.9×10^{-1}	8.2×10^{-1}	0.19	0.01	0.29	0.09
04/07/11	T2	NASE	26.11	−35.27	25.01	37.63	50.86	6.3×10^{-1}	n.d.	9.6×10^{-2}	7.3×10^{-1}	0.05	0.01	0.28	0.08
06/07/11	T2	NASE	27.98	−29.67	22.77	37.32	50	n.d.	n.d.	4.2	4.2	0.03	0.00	0.41	0.06
09/07/11	T2	NASE	30.96	−20.64	20.96	36.88	41.88	6.5×10^{-1}	n.d.	7.1×10^{-1}	1.4	0.06	0.00	0.45	0.10
10/07/11	T2	NASE	32.09	−17.26	21.37	36.69	38.5	5.8×10^{-1}	n.d.	1.3×10^{-2}	5.9×10^{-1}	0.05	0.00	0.44	0.09
16/12/10	T3	NASE	35.20	−9.56	19.17	36.56	94.5	$5.0 \times 10^{-5} \pm 7.1 \times 10^{-5}$	$1.6 \times 10^{-5} \pm 2.3 \times 10^{-8}$	$2.2 \times 10^{-5} \pm 3.1 \times 10^{-5}$	$8.8 \times 10^{-5} \pm 3.6 \times 10^{-5}$	0.26	-	0.49	0.08
17/12/10	T3	NASE	34.00	−12.78	19.87	36.56	107.2	$1.7 \times 10^{-4} \pm 1.5 \times 10^{-4}$	$8.0 \times 10^{-5} \pm 8.5 \times 10^{-5}$	$8.6 \times 10^{-5} \pm 8.4 \times 10^{-5}$	$3.4 \times 10^{-4} \pm 3.2 \times 10^{-4}$	-	-	-	-
19/12/10	T3	NASE	29.70	−17.28	21.29	36.92	101.5	$8.3 \times 10^{-5} \pm 2.8 \times 10^{-5}$	$2.3 \times 10^{-5} \pm 1.1 \times 10^{-5}$	$3.6 \times 10^{-5} \pm 2.0 \times 10^{-5}$	$1.4 \times 10^{-4} \pm 5.9 \times 10^{-5}$	0.12	-	-	-
21/12/10	T3	NASE	24.97	−21.06	23.25	37.00	103.6	$1.6 \times 10^{-3} \pm 8.8 \times 10^{-4}$	$4.1 \times 10^{-5} \pm 3.3 \times 10^{-6}$	$1.0 \times 10^{-4} \pm 2.8 \times 10^{-6}$	$1.7 \times 10^{-3} \pm 8.7 \times 10^{-4}$	-	-	0.73	0.01
22/12/10	T3	NATR	23.23	−22.26	24.04	37.07	542.5	$4.1 \times 10^{-4} \pm 1.8 \times 10^{-4}$	$7.8 \times 10^{-6} \pm 2.9 \times 10^{-6}$	$2.6 \times 10^{-5} \pm 1.2 \times 10^{-5}$	$4.5 \times 10^{-4} \pm 1.9 \times 10^{-4}$	0.23	-	0.62	0.02
23/12/10	T3	NATR	21.43	−23.46	25.09	36.97	481	$1.1 \times 10^{-3} \pm 1.4 \times 10^{-3}$	$6.7 \times 10^{-6} \pm 5.6 \times 10^{-7}$	$3.6 \times 10^{-5} \pm 3.0 \times 10^{-5}$	$1.1 \times 10^{-3} \pm 1.5 \times 10^{-3}$	0.18	-	-	0.08
24/12/10	T3	NATR	20.26	−24.36	25.68	36.71	252.8	$7.0 \times 10^{-5} \pm 4.0 \times 10^{-5}$	$2.9 \times 10^{-5} \pm 2.9 \times 10^{-5}$	$6.3 \times 10^{-5} \pm 5.6 \times 10^{-5}$	$1.6 \times 10^{-4} \pm 1.3 \times 10^{-4}$	0.14	-	-	-
25/12/10	T3	NATR	16.14	−26.00	26.60	36.40	244.5	$5.6 \times 10^{-5} \pm 1.3 \times 10^{-5}$	$1.0 \times 10^{-5} \pm 1.5 \times 10^{-6}$	$1.7 \times 10^{-5} \pm 5.5 \times 10^{-6}$	$8.3 \times 10^{-5} \pm 1.7 \times 10^{-5}$	0.21	-	0.21	-
26/12/10	T3	NATR	14.52	−26.01	26.80	36.27	109.2	$6.2 \times 10^{-5} \pm 8.8 \times 10^{-5}$	n.d.	$1.7 \times 10^{-5} \pm 2.5 \times 10^{-5}$	$7.9 \times 10^{-6} \pm 6.4 \times 10^{-5}$	0.26	-	0.47	0.04
27/12/10	T3	NATR	12.51	−26.04	27.49	35.63	112	$8.9 \times 10^{-5} \pm 1.3 \times 10^{-4}$	n.d.	$1.4 \times 10^{-3} \pm 2.0 \times 10^{-3}$	$1.5 \times 10^{-3} \pm 1.8 \times 10^{-3}$	0.26	-	0.07	0.03
28/12/10	T3	WTRA	9.56	−26.00	28.00	35.38	113.4	$2.0 \times 10^{-4} \pm 1.3 \times 10^{-4}$	n.d.	$1.2 \times 10^{-4} \pm 8.0 \times 10^{-5}$	$3.2 \times 10^{-4} \pm 4.7 \times 10^{-5}$	0.22	-	0.53	0.02
29/12/10	T3	WTRA	7.32	−26.00	28.33	35.41	106.4	$7.6 \times 10^{-4} \pm 9.3 \times 10^{-4}$	$2.9 \times 10^{-5} \pm 4.1 \times 10^{-5}$	$8.1 \times 10^{-5} \pm 6.3 \times 10^{-6}$	$8.7 \times 10^{-4} \pm 8.8 \times 10^{-4}$	0.25	-	0.34	0.02
30/12/10	T3	WTRA	5.01	−26.03	28.54	35.53	117.2	$1.7 \times 10^{-4} \pm 6.6 \times 10^{-5}$	n.d.	$8.4 \times 10^{-5} \pm 2.9 \times 10^{-5}$	$2.5 \times 10^{-4} \pm 3.7 \times 10^{-5}$	0.44	-	0.17	0.03
31/12/10	T3	WTRA	2.47	−26.03	28.10	35.69	132.8	$2.5 \times 10^{-5} \pm 3.5 \times 10^{-5}$	n.d.	$7.4 \times 10^{-6} \pm 1.0 \times 10^{-5}$	$3.2 \times 10^{-5} \pm 2.5 \times 10^{-5}$	0.22	-	-	-
01/01/11	T3	WTRA	0.24	−26.02	27.49	36.10	114.8	$4.0 \times 10^{-3} \pm 5.6 \times 10^{-3}$	n.d.	$1.9 \times 10^{-3} \pm 2.7 \times 10^{-3}$	$6.0 \times 10^{-3} \pm 8.3 \times 10^{-3}$	0.70	-	-	-
02/01/11	T3	WTRA	−3.03	−27.33	28.07	36.17	112	$8.0 \times 10^{-5} \pm 2.0 \times 10^{-5}$	n.d.	$5.1 \times 10^{-5} \pm 3.4 \times 10^{-5}$	$1.3 \times 10^{-4} \pm 1.4 \times 10^{-5}$	0.15	-	-	-
03/01/11	T3	WTRA	−4.78	−28.17	26.99	37.30	120	n.d.	n.d.	$7.1 \times 10^{-5} \pm 1.0 \times 10^{-4}$	$7.1 \times 10^{-5} \pm 1.0 \times 10^{-4}$	0.11	-	-	0.15
04/01/11	T3	SATL	−7.22	−29.34			125	n.d.	n.d.	$1.3 \times 10^{-5} \pm 1.4 \times 10^{-5}$	$1.3 \times 10^{-5} \pm 1.4 \times 10^{-5}$	0.10	-	0.08	0.13
05/01/11	T3	SATL	−9.12	−30.19	27.88	36.67	112	n.d.	n.d.	n.d.	n.d.	0.06	-	0.08	0.21
06/01/11	T3	SATL	−11.59	−31.40	27.76	36.88	113.4	n.d.	n.d.	n.d.	n.d.	0.04	-	0.40	0.14

Table S1. *Cont.*

07/01/11	T3	SATL	−13.73	−32.38	27.60	37.12	110.6	n.d.	n.d.	n.d.	n.d.	0.05	-	-	0.13
08/01/11	T3	SATL	−15.83	−33.41	27.89	37.22	113.4	n.d.	n.d.	n.d.	n.d.	0.09	-	-	0.13
09/01/11	T3	SATL	−18.40	−34.68	27.81	37.25	110.6	$1.9 \times 10^{-3} \pm 8.7 \times 10^{-5}$	n.d.	n.d.	$1.9 \times 10^{-3} \pm 8.7 \times 10^{-5}$	0.11	-	1.38	0.14
10/01/11	T3	SATL	−21.09	−35.98	27.53	37.01	112	$3.6 \times 10^{-3} \pm 5.1 \times 10^{-3}$	n.d.	$3.0 \times 10^{-5} \pm 4.2 \times 10^{-5}$	$3.6 \times 10^{-3} \pm 5.1 \times 10^{-3}$	0.13	-	1.59	0.11
11/01/11	T3	SATL	−22.99	−36.98	26.87	36.60	113.4	$9.4 \times 10^{-5} \pm 5.8 \times 10^{-5}$	n.d.	$7.0 \times 10^{-5} \pm 4.3 \times 10^{-5}$	$1.6 \times 10^{-4} \pm 1.0 \times 10^{-4}$	-	-	1.06	0.07
19/01/11	T4	SATL	−24.32	−36.22	27.05	36.37	116.2	n.d.	n.d.	n.d.	n.d.	0.08	-	0.14	0.03
20/01/11	T4	SATL	−24.81	−33.46	27.05	36.54	135.35	n.d.	n.d.	n.d.	n.d.	0.09	-	0.27	0.01
21/01/11	T4	SATL	−25.43	−30.07	26.13	36.56	127.3	$5.2 \times 10^{-4} \pm 5.5 \times 10^{-4}$	n.d.	$3.4 \times 10^{-5} \pm 4.8 \times 10^{-5}$	$5.5 \times 10^{-4} \pm 5.0 \times 10^{-4}$	0.10	-	0.35	0.00
22/01/11	T4	SATL	−25.87	−27.57	25.61	36.43	113.5	$8.1 \times 10^{-5} \pm 9.2 \times 10^{-7}$	n.d.	$4.2 \times 10^{-5} \pm 9.0 \times 10^{-6}$	$1.2 \times 10^{-4} \pm 8.1 \times 10^{-6}$	0.08	-	0.00	0.11
23/01/11	T4	SATL	−26.46	−24.21	24.65	36.33	115	$3.5 \times 10^{-4} \pm 3.4 \times 10^{-4}$	$6.6 \times 10^{-5} \pm 1.1 \times 10^{-5}$	$1.0 \times 10^{-4} \pm 1.9 \times 10^{-5}$	$5.2 \times 10^{-4} \pm 3.7 \times 10^{-4}$	0.05	-	0.34	0.06
24/01/11	T4	SATL	−26.94	−21.40	24.27	36.24	114.75	$5.1 \times 10^{-4} \pm 5.0 \times 10^{-4}$	$6.3 \times 10^{-5} \pm 2.2 \times 10^{-5}$	$6.4 \times 10^{-5} \pm 9.1 \times 10^{-5}$	$6.3 \times 10^{-4} \pm 4.3 \times 10^{-4}$	0.10	-	0.20	0.10
25/01/11	T4	SATL	−27.55	−18.09	24.16	36.33	124.05	n.d.	$8.4 \times 10^{-5} \pm 9.3 \times 10^{-5}$	$8.0 \times 10^{-5} \pm 9.7 \times 10^{-5}$	$1.6 \times 10^{-4} \pm 1.9 \times 10^{-4}$	0.04	-	0.10	0.10
26/01/11	T4	SATL	−28.13	−14.78	24.05	36.49	114.1	n.d.	$5.7 \times 10^{-2} \pm 8.1 \times 10^{-2}$	$4.8 \times 10^{-5} \pm 6.8 \times 10^{-5}$	$5.7 \times 10^{-2} \pm 8.1 \times 10^{-2}$	0.04	-	0.38	0.11
27/01/11	T4	SATL	−28.65	−11.81	23.95	36.38	114.8	n.d.	$5.2 \times 10^{-2} \pm 7.4 \times 10^{-2}$	$5.9 \times 10^{-2} \pm 8.3 \times 10^{-2}$	$1.1 \times 10^{-1} \pm 1.6 \times 10^{-1}$	0.04	-	0.29	0.14
28/01/11	T4	SATL	−29.10	−9.14	23.19	36.08	118.25	n.d.	$2.8 \times 10^{-2} \pm 3.2 \times 10^{-2}$	n.d.	$2.8 \times 10^{-2} \pm 3.2 \times 10^{-2}$	-	-	-	-
29/01/11	T4	SATL	−29.77	−5.31	22.95	36.06	122.55	n.d.	$7.8 \times 10^{-3} \pm 6.6 \times 10^{-4}$	n.d.	$7.8 \times 10^{-3} \pm 6.6 \times 10^{-4}$	0.04		0.33	0.15
30/01/11	T4	SATL	−30.26	−2.44	22.98	35.98	135.9	$1.1 \times 10^{-2} \pm 1.5 \times 10^{-2}$	$1.3 \times 10^{-2} \pm 1.7 \times 10^{-2}$	n.d.	$2.4 \times 10^{-2} \pm 3.2 \times 10^{-2}$	0.04		0.36	0.17
31/01/11	T4	SATL	−30.88	0.97	22.15	35.94	120.05	$1.7 \times 10^{-3} \pm 2.2 \times 10^{-3}$	$6.6 \times 10^{-3} \pm 9.1 \times 10^{-3}$	n.d.	$8.3 \times 10^{-3} \pm 1.1 \times 10^{-2}$	0.06		0.36	0.18
01/02/11	T4	SATL	−31.31	3.75	21.69	35.80	114.45	$5.0 \times 10^{-3} \pm 2.4 \times 10^{-3}$	$7.1 \times 10^{-2} \pm 3.5 \times 10^{-3}$	n.d.	$7.6 \times 10^{-2} \pm 3.3 \times 10^{-2}$	0.05		0.34	0.11
02/02/11	T4	SATL	−31.83	6.86	21.33	35.79	115.15	$1.2 \times 10^{-3} \pm 1.5 \times 10^{-3}$	$5.9 \times 10^{-3} \pm 7.0 \times 10^{-3}$	n.d.	$7.2 \times 10^{-3} \pm 8.5 \times 10^{-3}$	0.06		0.00	0.13
03/02/11	T4	BENG	−32.22	9.35	21.35	35.73	117.6	n.d.	$7.9 \times 10^{-2} \pm 8.0 \times 10^{-3}$	n.d.	$7.9 \times 10^{-2} \pm 8.0 \times 10^{-3}$	0.07		0.30	0.08
04/02/11	T4	BENG	−32.81	12.77	21.11	35.49	115.85	$2.1 \times 10^{-3} \pm 2.9 \times 10^{-3}$	$1.6 \times 10^{-2} \pm 2.2 \times 10^{-2}$	$1.8 \times 10^{-5} \pm 2.6 \times 10^{-5}$	$1.8 \times 10^{-2} \pm 2.5 \times 10^{-2}$	0.07		0.52	0.14
05/02/11	T4	BENG	−33.23	15.34	20.43	35.42	117.6	$2.5 \times 10^{-3} \pm 2.3 \times 10^{-3}$	$3.2 \times 10^{-1} \pm 7.0 \times 10^{-2}$	n.d.	$3.2 \times 10^{-1} \pm 7.3 \times 10^{-2}$	0.23		0.14	0.12

© 2014 by the authors; licensee MDPI, Basel, Switzerland. This article is an open access article distributed under the terms and conditions of the Creative Commons Attribution license (http://creativecommons.org/licenses/by/3.0/).
